# A single-nucleus multiomic study of preoptic area cells in prepubertal control vs prenatally androgenized female mice

**DOI:** 10.1210/endocr/bqag087

**Published:** 2026-08-01

**Authors:** Laura L Burger, Rujuta M Chikodikar, Suzanne M Moenter

**Affiliations:** Department of Molecular and Integrative Physiology, University of Michigan, Ann Arbor, MI 48109, USA; Department of Molecular and Integrative Physiology, University of Michigan, Ann Arbor, MI 48109, USA; Department of Molecular and Integrative Physiology, University of Michigan, Ann Arbor, MI 48109, USA; Department of Internal Medicine, University of Michigan, Ann Arbor, MI 48109, USA; Department of Obstetrics and Gynecology, University of Michigan, Ann Arbor, MI 48109, USA; Department of Reproductive Sciences Program, University of Michigan, Ann Arbor, MI 48109, USA

**Keywords:** PMOS, PCOS, prepubertal, reproduction, neuroendocrinology

## Abstract

Polycystic ovary syndrome (PCOS) is a common endocrine disorder with developmental origins. While the etiology is unclear, current postulates include epigenetic programming. Cell-type-specific epigenetic changes by which prenatal androgen excess programs the neuroendocrine axis have not been defined. Using single-nucleus (sn) multiome sequencing (single-nucleus ribonucleic acid sequencing [snRNAseq] + single-nucleus assay for transposase-accessible chromatin using sequencing [snATACseq]) of the mouse preoptic area, we profiled transcriptional and chromatin accessibility landscapes across 31 cell populations on postnatal day 18 to 22 in a prenatal androgenization (PNA) mouse model that produces neuroendocrine phenotypes that resemble hyperandrogenemic PCOS. Marker gene analysis identified 17 neuronal and 14 non-neuronal populations. We refined the gonadotropin-releasing hormone (GnRH) neuron cluster to 41 neurons by manual curation. Cross-dataset comparisons were used to characterize the molecular transcriptional identity of these clusters. Gene set enrichment analysis of mRNA expression data revealed enrichment of protein synthesis and oxidative phosphorylation pathways and suppression of TNF/NF-κB signaling and steroid responsiveness across several clusters in PNA animals. Pseudobulk differential chromatin accessibility testing across ∼30 600 peaks identified 15 false discovery rate-significant differentially accessible regions, including 2 loci in GnRH neurons at genomic regions of unknown function, suggesting prenatal androgen exposure changes chromatin accessibility in this and other cell types. Chromosome accessibility at most sex steroid receptor genes was surprisingly present in GnRH neurons. Reduced *Pgk1* promoter accessibility in multiple glial populations suggests PNA alters epigenetic regulation of glial energy metabolism. These findings support a model of developmental programming in which prenatal androgen exposure produces cell-type-specific changes that include, but are not limited to, epigenetic remodeling to generate the PNA phenotype.

The hypothalamic-pituitary-gonadal axis controls reproduction in vertebrates. Gonadotropin-releasing hormone (GnRH) neurons in the medial preoptic area (POA) and hypothalamus project to the median eminence, releasing GnRH in discrete pulses in males and during most of the female reproductive cycle (1-5). GnRH induces the synthesis and release of the gonadotropins, luteinizing hormone (LH) and follicle-stimulating hormone (FSH), by the anterior pituitary (6). The release occurs in a GnRH pulse-frequency-dependent manner, with high frequencies favoring LH release and low frequencies favoring FSH release (7, 8). Gonadotropins initiate the synthesis of sex steroids, which feedback to regulate the brain and pituitary (9). Disruptions in this axis can lead to reduced fertility. Polycystic ovary syndrome (PCOS, which was recently renamed polyendocrine metabolic ovary syndrome, PMOS (10)) affects 10% to 13% of reproductive-aged women (11) according to the Rotterdam diagnostic criteria (12) and is the leading cause of female infertility (13, 14). Hyperandrogenemic PCOS, affecting 8% to 20% of reproductive-aged women (6, 14), is associated with persistent high-frequency LH, and presumably GnRH, pulses (6) and a higher LH:FSH ratio compared to healthy women (15). These disruptions likely begin during the pubertal process; adolescent girls with hyperandrogenemia exhibit elevated LH-pulse frequency (16).

The underlying causes of PCOS are not fully understood. PCOS is highly heritable with 20% to 40% of first-degree relatives of women with PCOS developing this disorder (17, 18). Current understanding postulates that PCOS is multifactorial, with genetics and environment both contributing to its etiology. In terms of genetics, genome-wide association studies have linked single nucleotide polymorphisms in over 20 genes to PCOS (19-22). These changes account for <10% of cases, however, suggesting the presence of additional contributing factors (23). When considering environmental factors, pregnant women with PCOS have elevated serum testosterone levels in the latter part of gestation (24, 25), suggesting that fetal exposure to maternal androgens may drive development of PCOS (21, 26). Supporting this, prenatal androgenization (PNA) of mice, rats, sheep, and non-human primates produces female offspring that display a PCOS-like phenotype: elevated serum LH and testosterone (27-32) and elevated LH-pulse frequency (33-35), providing substantial evidence that PNA induces a reprograming of the hypothalamic-pituitary-gonadal axis. Transgenerational transmission of PCOS-like traits was recently reported in both PNA and anti-Müllerian hormone excess models (36-38), underscoring the importance of identifying epigenetic events before adult phenotypes emerge.

To determine the effects of PNA on the transcriptional profile of GnRH neurons in mice, translating ribosome affinity purification combined with ribonucleic acid sequencing (TRAP-seq) was performed on female vehicle-control treated (VEH) and PNA dams (39). TRAP-seq revealed that PNA altered the transcriptional profile of GnRH neurons. We postulate that as an underlying mechanism, PNA treatment induces epigenetic changes in GnRH neurons. Here, the epigenetic and nuclear-RNA expression profiles of GnRH neurons and surrounding cells were identified in prepubertal mice in an unbiased manner using multiomics: a combination of single-nucleus assay for transposase-accessible chromatin using sequencing (snATACseq) and single-nucleus ribonucleic acid sequencing (snRNAseq). The goals were to determine the epigenetic and gene expression (GEX) profiles of GnRH neurons in VEH vs PNA female mice, and to determine the changes in epigenetic and GEX profiles of non-GnRH cells in the preoptic area to gain insight into the mechanisms underpinning PNA-induced changes in GEX that occur before the onset of adult differences in phenotype arising from that treatment, such as elevated androgens in PNA mice.

## Materials and methods

Reagents were obtained from MilliporeSigma unless noted.

### Animals

Mice were provided with water and Harlan 2916 chow *ad libitum* and were housed on a 14L:10D light cycle with lights on at 0300 Eastern Standard Time. Male mice on a C57Bl6/J background and homozygous for GnRH-cre (JAX 021207) (40) were mated with 2 female mice: one female (C57Bl6/J background) homozygous for R26-loxSTOPlox-Sun1-green fluorescent protein (GFP) (JAX 021039) (41) to generate Sun1-GFP labeled nuclei in GnRH neurons, and one wild-type CD1 female (Charles River Laboratories, Wilmington, USA) for maternal and nutritional support (42). Although GFP-assisted nuclear isolation ultimately failed due to nonspecific binding, these mice were thus used for unbiased nuclear isolation to avoid animal waste. Females were checked daily for a copulatory plug, and males were removed once pregnancy was established. On days 16, 17, and 18 of gestation, Sun1-GFP females were injected subcutaneously either with 225 µg of dihydrotestosterone (DHT) (50 µL of 4.5 µg/µL DHT in 90% sesame oil/10% ethanol; PNA group) to induce PNA (*n* = 4) or a control vehicle (VEH group, *n* = 5). Pups were genotyped at postnatal day (PND)14 for Cre and GFP. Pups from the CD1 dam, distinguished by coat color, were culled to maintain <15 pups per cage. All animal work was approved by the Institutional Animal Care and Use Committee of the University of Michigan (PRO00012163).

### Tissue collection

Preoptic area tissue was collected from female VEH (18 mice from 5 litters) and PNA (12 mice from 4 litters) pups at age 18 to 22 days. Mice were deeply anesthetized with isoflurane. Brains were removed and placed in ice-cold sucrose–saline solution (250 mM sucrose [Invitrogen/ThermoFisher, Waltham, MA, USA], 26 mM NaHCO_3_, 1.25 mM NaHPO_4_, 1.2 mM MgSO_4_, 10 mM D-glucose [Invitrogen/ThermoFisher], 3.5 mM KCl, 2.5 mM MgCl_2_) for 15 seconds. Each brain was positioned in an ice-cold 1-mm coronal brain matrix (Zivic Instruments, Pittsburgh, PA, USA). A coronal brain slice extending 2 mm rostral from the caudal extent of the optic chiasm was obtained, and a 1.2 mm diameter Palkovits punch was utilized to obtain a punch of tissue at the ventral extent of the tissue on midline (volume ∼2.26 mm^3^). Tissue punches from all female mice in a litter were combined, placed in a microcentrifuge tube on dry ice during collection of tissue from the remaining mice in the litter, then transferred to liquid nitrogen, and stored at −80 °C until nuclear isolation.

### Nuclear isolation

The 10X Genomics Chromium Controller (10X Genomics, Pleasanton, CA) can process 8 samples on a single microfluidics chip. We thus split the nucleus isolation of the 9 total samples (5 VEH and 4 PNA) over 2 days. Four samples were chosen at random on day 1, and the remainder were processed on day 2. The protocol for nuclear isolation was derived from multiple sources (41, 43, 44). Each sample was transferred to a 2-mL glass Dounce homogenizer (Kimble Kontes/DWK Life Science, Millville, NJ, USA) containing 350 µL of ice-cold homogenization buffer (250 mM sucrose [Invitrogen/ThermoFisher]), 20 mM Tricine-KOH pH 7.5, 5 mM MgCl_2_ (Applied Biosystems/Life Technologies, Waltham, MA, USA), 25 mM KCl (Applied Biosystems/Life Technologies), 0.015 mM spermine, 0.5 mM spermidine, 1 mM DTT, 1 × complete mini EDTA-free protease inhibitor cocktail tablets, 15 U/mL Protector RNase inhibitor, nuclease-free water (Invitrogen/ThermoFisher). The tissue was homogenized using 15 to 18 strokes of pestle A (clearance = 0.0025-0.0055 inches), followed by the addition of 11 µL of 10% NP-40 (0.30% final concentration, ThermoFisher Scientific). Next, homogenization with 10 strokes of pestle B (clearance = 0.0005-0.0025 inches) was performed, followed by filtration with a 40 µM Flowmi filter (Bel Art, Wayne, NJ, USA) and a subsequent centrifugation at 500*×g* for 10 minutes at 4 °C in a swinging bucket microcentrifuge to pellet the nuclei. The pellet was resuspended in 475 µL of Tricine-BSA (150 mM KCl, 30 mM MgCl_2_, 120 mM Tricine, pH 7.5, 1 mM DTT, 15 U/mL Protector RNase Inhibitor, 1 × mini EDTA-free protease inhibitor cocktail tablets, 0.05% bovine serum albumin [Jackson ImmunoResearch, West Grove, PA, USA]). Next, 25 µL of magnetically labeled anti-nucleus MicroBeads (Miltenyi Biotec, Auburn, CA, USA) were added to positively select intact nuclei and allow removal of cellular debris, and the mixture was incubated end-over-end for 1 hour at 4 °C. A miniMACS Separator column (Miltenyi Biotec) was placed in a magnetic MACS Separator (Miltenyi Biotec) and prerinsed with 500 µL of Tricine-BSA, the nuclei sample was added and the effluent was discarded. Columns were washed twice with 500 µL Tricine-BSA and then removed from the magnet. A volume of 50 µL of 1 × nuclei buffer (diluted 20 × nuclei buffer from 10X Genomics, Pleasanton, CA, USA) was added to flush the nuclei from the column. A final centrifugation at 1000*×g* for 10 minutes at 4 °C was performed to pellet the nuclei and the volume was drawn down to ∼20 µL. Samples were then submitted to the University of Michigan Advanced Genomics Core for microfluidics to form gel beads in emulsion, library preparation, and sequencing.

To determine accurate targeting of the POA punch, 3 µL of the nuclei suspension was held back to determine *Gnrh1* mRNA expression in the nuclei. Using TaqMan Cells-to-CT Express Kit (Invitrogen/ThermoFisher), nuclei were lysed, RNA was treated with DNase (deoxyribonuclease), reverse-transcribed to cDNA and amplified by TaqMan qPCR for *Gnrh1*, *Actb*, and *Fabp2*. The *Fabp2* amplicon is in the promoter/exon1 region of the mouse DNA and was used to ensure that there was no genomic DNA remaining in the samples. *Gnrh1* mRNA was normalized to *Actb* mRNA (housekeeping transcript) and *Gnrh1* enrichment is expressed as fold increase vs whole hypothalamic mRNA. *Gnrh1* mRNA expression was similarly enriched in VEH (5.4 ± 0.8-fold) and PNA (4.6 ± 0.4-fold) samples compared to whole POA.

### Sequencing

The nuclei were processed for 10X Genomics Multiome ATAC (assay for transposase-accessible chromatin) + GEX. Nuclei preparations were counted on the LunaFx7 automated cell counter (Logos Biosystems, Annandale, VA, USA) using acridine orange and propidium iodide staining and diluted, if necessary, to ∼1200-5000 nuclei/µL. Nuclei were transposed in bulk to fragment accessible chromatin and add adapters. Nuclei were then encapsulated in microfluidic droplets (gel beads in emulsion) by the Chromium Controller. Each bead contains unique barcodes, which tag both ATAC and GEX products to a specific nucleus, and reverse transcriptase reagents. Following encapsulation and reverse transcription, the droplets were dissolved and the bar-coded tagmented DNA and cDNA were isolated, per 10X Genomics' recommendations, and processed for library construction. Quality of each library was assessed using LabChip GX Touch HT (Revvity, Waltham, MA, USA). Both libraries were quantified by Qubit (Invitrogen/ThermoFisher) and assessed for size on the TapeStation 4200 (Agilent Technologies, Santa Clara, CA, USA). Pooled libraries were subjected to 50 000 reads/cell paired-end sequencing according to the manufacturer's protocol (Illumina NovaSeqx Plus). BclConvert software (Illumina, San Diego, CA, USA) was used to generate de-multiplexed Fastq files, and the CellRanger Arc (version 2.0.2) Pipeline (10X Genomics) was used to align reads to the mm10 mouse genome and generate count matrices (45). The Cell Ranger filtered barcode feature matrix was used as input to downstream analysis. One VEH sample (V5) suffered a major microfluidic clog during bead emulsion and most of the sample was lost and not sequenced; a PNA sample (P7) had a minor microfluidic clog, but sufficient nuclei were recovered and sequencing continued. The final sets of samples were 4 biological replicates from each treatment group.

### UMAP analyses

Initial single nuclei ATAC and GEX sequencing analyses were done by the University of Michigan Bioinformatics Core using R (v4.1.3) (46) primarily using the Seurat package for GEX (v4.1.0) (47) and the Signac R package for ATAC (v1.6.0). We applied quality-control filters: nCount_ATAC >1000 (minimum of 1000 unique accessible chromatin fragments/nuclei), nCount_RNA > 1000 (minimum 1000 unique molecular identifiers for GEX per nuclei) and TSS.enrichment >3 (transcription start site enrichment score >3, indicating good ATAC-seq quality). This resulted in 51 282 total nuclei (6404 ± 852, *n* = 8 samples) after filtering. These thresholds eliminated extreme values, which might indicate low complexity, doublets, or damaged or apoptotic nuclei, but ensured adequate coverage depth of both data types. To identify potential heterotypic doublets, scDblFinder (v1.16, Bioconductor) (48) was applied to the full, quality-control filtered dataset (51 282 nuclei) using default parameters and SerialParam execution to improve reproducibility. Flags for putative doublet nuclei were generated for each sample, since doublet formation is primarily a function of sample nuclear loading density.

Following quality control, the ATAC peaks were re-called with MACS (version 2) (49), normalized via term frequency-inverse document frequency by Signac (50). GEX counts were normalized using the SCTransform method with default parameters by Seurat (51). The ATAC and GEX data were merged into a single Seurat object and checked to determine if isolating nuclei on different days and/or treatment resulted in any batch effect by visualizing nuclei on Uniform Manifold Approximation and Projection (UMAP) plots colored by both sample of origin and treatment group. Neighborhood mixing was evaluated by computing the Local Inverse Simpson Index (LISI) for each nucleus using the weighted-nearest-neighbor (WNN) UMAP embedding (LISI R package) (52). For the treatment covariate (VEH vs PNA, *n* = 2) the mean LISI score was 1.838 out of a theoretical maximum of 2.0 indicating that both treatments share the same biological space and do not segregate into treatment-specific artifacts. The mean LISI score for the sample covariate was 4.721 (V1-4 and PNA6-9, *n* = 8); as the design contains 4 technical replicates per treatment a LISI score of 4.721 out of 8 indicates that the replicates are well integrated within their biological treatment. Of note, integration tools such as Harmony carry the risk of misidentifying rare cell types as batch variance and collapsing them back into neighboring cells (53), which is at odds with the goal of identifying the very sparse GnRH neuron population. Given the high degree of sample and treatment mixing, therefore, formal integration was omitted and simple merging was used to preserve true biological variance and prevent overcorrection (Fig. S1, Table S1) (54).

Clustering was done using the GEX data by dimensional reduction and visualization. Several combinations of principal components (PCs, 10-50) and clustering resolutions (0.1-1.0) were tested and evaluated for both separation of *Gnrh1*-expressing nuclei and overall cluster structure. We selected 25 PCs and resolution 0.25 as this combination provided distinct separation of the GnRH neuron population while maintaining biologically interpretable clustering without over-clustering across all cell types. Similar dimensional reduction, via singular value decomposition, to 25 components and resolution 0.25 was done on the ATAC data. UMAP plots were generated using default settings (55), which resulted in 34 clusters (0-33).

### Marker gene analyses

To identify marker genes for clusters that were consistent across treatment groups, clusters were compared pairwise with all other clusters for differential GEX using the Wilcoxon rank-sum test via the FindConservedMarkers() function in Seurat. This approach identifies genes that are markers of a cluster in both PNA and VEH conditions. Marker genes were defined as those expressed in ≥50% of nuclei within the cluster, with log2 fold change ≥0.585 (1.5-fold) vs other clusters, adjusted *P*-value <.05, and expression in <30% of nuclei outside the cluster. These marker gene lists underwent initial evaluation using Claude (Anthropic, Claude Sonnet 4.5) to identify likely cell-type identities based on known expression patterns in the literature. Proposed cell-type assignments were independently validated by a literature search, single-cell databases such as mousebrain.org (56) and HypoMap (57, 58). Neuronal clusters were screened for neuropeptide genes, the classical neurotransmitter system genes (*Gad1*, *Gad2*, *Slc17a6*, *Slc17a7*, etc.) and functional and/or specialized marker genes such as hormone receptors (eg, *Lepr, Esr1, Esr2, Crhr1*), processing enzymes (*Pcsk1, Cpe, Th, Gad1, Gad2*), and transcription factors involved in brain development (*Sim1, Dlx1, Lhx8*). The clusters were named as primary neuropeptide + classical neurotransmitter + functional context (if necessary). Three clusters were excluded from downstream analyses: clusters 26 and 28 consisted predominantly of nuclei from a single sample, suggesting sample-specific artifacts rather than biologically distinct cell types, and cluster 32 displayed mixed-cell-type marker expression. All 3 clusters are small (303, 183, and 52 nuclei, respectively) and contain only 1% of the total nuclei. The remaining 31 clusters were used for all subsequent analyses.

### Manual clustering of GnRH neurons

For GnRH neurons, 31 nuclei were originally grouped in cluster 33. There were 24 additional nuclei that expressed considerable *Gnrh1* (raw counts >5) but were not a part of cluster 33. To determine if these other *Gnrh1*-expressing nuclei were from authentic GnRH neurons, we examined a heatmap of the residuals from SCTransform among nuclei with raw *Gnrh1* counts > 5 and identified 12 additional nuclei with similar residual expression patterns in the top 3000 most variable genes. We thus created a manual GnRH neuron cluster nuclei referred to as m33.

### Differential gene analyses

For each cluster, pseudobulk differential expression analysis was performed by aggregating counts across nuclei of the same treatment group to increase statistical power. For both GEX and chromatin accessibility (ATAC) data, treatment effects were tested using pseudoBulkDGE() function from the scran package (v1.22.1) (59) with treatment as the predictor. The contrast between PNA and VEH conditions was evaluated, applying significance thresholds of nominal *P*-value < .05 and |log2FC| > 0.585. This analysis was performed for all clusters including both the manually curated GnRH cluster and the algorithmically generated GnRH population; both datasets are provided, but further analysis was restricted to the manually curated cluster.

For the peak-to-gene linkage, putative regulatory relationships between ATAC peaks and GEX were identified using Pearson correlation-based linkage analysis (60). Peak-to-gene linkage was conducted using the Signac defaults; for each gene-peak pair within ±50 kb of the TSS, Pearson correlation between chromatin accessibility and GEX was calculated across nuclei, with statistical significance assessed relative to an empirical null distribution generated from background peaks matched for chromatin accessibility and GC content (one-sided *z*-test, *P* < 0.05). To identify coordinated chromatin-transcription changes in response to treatment, peak-gene links were intersected with pseudobulk differential accessibility and expression results to identify linked pairs showing significant changes in both modalities (PNA vs VEH).

### Gene set enrichment analysis

Gene set enrichment analysis (GSEA) was performed in R using the fgseaMultilevel (fgsea v1.38.0) (61) with gene sets from MsigDB v2026.1 (msigdbr v7.5.1, msigdbrdata v26.1.0) to identify biological pathways enriched in each cell cluster. This approach facilitates identifying biologically relevant signals in smaller clusters where statistical power is limited (62, 63). For each cluster, all tested genes from the pseudobulk differential expression analysis were ranked by log2 fold change (+PNA to −VEH) without pre-filtering by significance to preserve the full distribution for enrichment testing. Ranked gene lists were tested against 6 curated gene set databases retrieved via the Molecular Signatures Database (MSigDB) package for *Mus musculus*: GO Biological Process, GO Molecular Function, GO Cellular Component, KEGG, Reactome, and MSigDB Hallmark gene sets (63, 64). Gene sets with fewer than 10 or more than 500 genes were excluded. GSEA was run with 10 000 permutations, and normalized enrichment score (NES) was assessed separately for PNA-enriched (positive NES) and VEH-enriched (negative NES) signatures. Results were filtered using a weighted composite score of NES × −log10(*P*_adj_) ≥ .903 (equivalent to approximately NES ≥ 1.5 at *P*_adj_ = .25), which recognizes strong enrichment and statistical confidence without imposing a hard threshold on either. GSEA is a hypothesis-generating tool and can be particularly susceptible to noise in small datasets.

All processed data, including quality-control metrics, marker gene lists, pseudobulk differential expression results, and gene ontology/GSEA results, are available via the University of Michigan Deep Blue Data repository (54) GEX and ATAC-seq data (raw and processed) were deposited in the NCBI GEO database under accession number GSE318722 (65).

Portions of the analysis code and manuscript text were developed with assistance from Claude (Anthropic, claude.ai). Specifically, Claude was used to assist with (1) cross-referencing ambiguous cluster marker gene lists against published hypothalamic cell-type databases and primary literature to aid in cluster identity assignment. Claude was not used for statistical analysis, data processing, or primary data interpretation. All AI-assisted cluster annotations were independently verified by the authors against HypoMap, mousebrain.org, and primary literature. All manuscript text was reviewed, revised as appropriate, and approved by all authors. Analysis code was reviewed and verified by the authors prior to use.

## Results

### Library quality control

**Figure 1:**
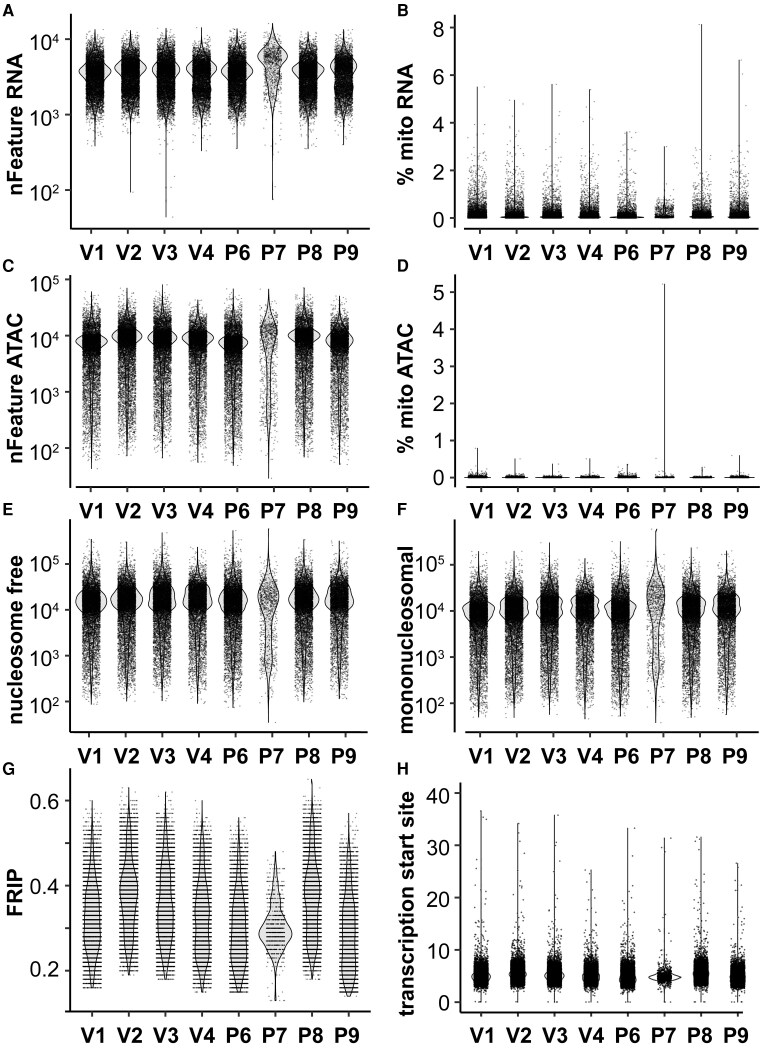
Following quality control, data were normalized and dimension reduction was conducted using the RNA modality and tracking *Gnrh1* expression. P7 is the sample that had the microfluidic clog, thus fewer nuclei.

Single-nucleus multiome libraries were sequenced to a combined depth of approximately 9.2 billion paired-end reads (∼4.0 billion GEX + ∼5.2 billion ATAC) across 8 biological samples. After nucleus-level quality control, 51 282 nuclei were retained (∼92% of the original). Following quality filtering (mapping quality >30 and removal of mitochondrial reads and PCR duplicates), approximately 1 billion unique ATAC fragments and 571 million RNA unique molecular identifiers remained. Quality metrics of the post-quality-control samples are shown in Fig. 1. The median number of genes per nuclei (nFeature) in the GEX assay was 3441 (range = 3328-5298, Fig. 1A). The median number of peaks in the ATAC assay (nFeatureATAC) was 18 364 (range = 10,547-25,281, Fig. 1C). The percentage of mitochondrial sequence was low across all samples (Fig. 1B and 1D) and nucleosome patterns (Fig. 1E and 1F) and ATAC-seq quality metrics (Fig. 1G and 1H) were comparable. For full quality-control data, see Table S1 (54). scDblFinder flagged 5889 of 51 282 nuclei (11.5%) as putative doublets across the dataset. Each nucleus is annotated with a scDblFinder.score and is identified as a singlicate or possible doublet in the metadata in Table S1 (54). The scDblFinder predicted doublet rates varied by sample in proportion to how many nuclei were loaded per sample (*r* = 0.992), as expected if doublets form largely by chance during microfluidic encapsulation. In contrast, doublet flagging frequency was variable and influenced by cluster identity. Cluster-specific doublet rates ranged from a low of 1.6% in quiescent oligodendrocyte precursor cells (Cluster 7) to a high of 96% in specialized astrocytes (Cluster 21), despite lower complexity (n_count RNA or ATAC) in cluster 21 “doublets,” suggested this high rate of doublet flagging is erroneous. The algorithmic GnRH neuron population (Cluster 33) exhibited a 0% doublet rate. Of note, κ-nearest neighbor (κ NN) graph-based doublet detection methods, including scDblFinder, perform less reliably in rare cell populations with limited same-cluster reference examples (66), therefore, per-cluster doublet burden must be evaluated on its own biological merits.

### Uniform Manifold Approximation Projections

**Figure 2:**
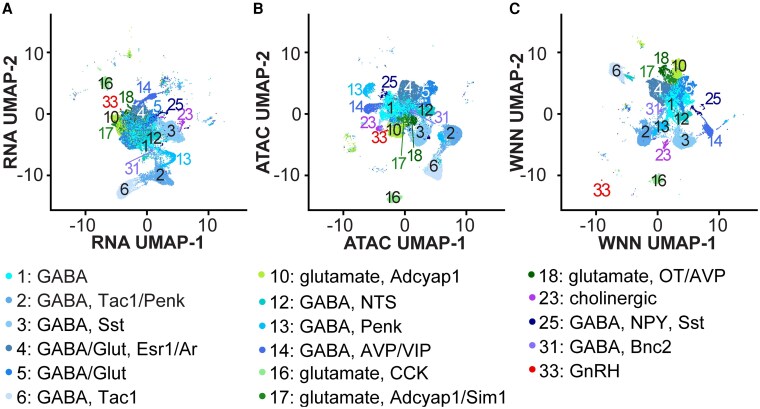
UMAP plots for snRNA (left), snATAC (middle), and combined weighted-nearest neighbor (WNN, right) for neuronal elements.

**Figure 3:**
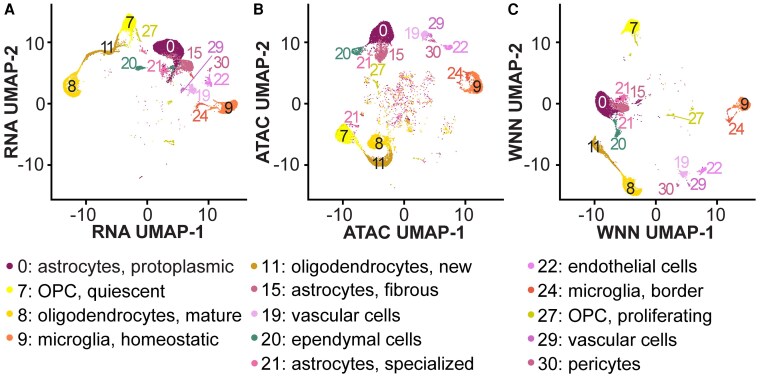
UMAP plots for snRNA (left), snATAC (middle), and combined weighted-nearest neighbor (WNN, right) for non-neuronal elements.

Uniform Manifold Approximation Projections were created for the RNA, ATAC, and combination of the modalities by WNN for 25 PCs at a resolution of 0.25. This combination resolved *Gnrh1-*expressing nuclei (cluster 33) without generating unnecessarily high numbers of clusters. Examination of the composition of the 34 clusters generated identified 3 problematic clusters; clusters 26 and 28 were composed primarily of nuclei from a single sample (VEH-3 and PNA-7, respectively) and cluster 32 showed a mixed-cell typed identification (Table 1). Across the entire data set the per-nucleus LISI scores was below 2 in just 760 nuclei (1.5% of the dataset); 61.4% of these low-LISI nuclei belonged to clusters 26 and 28 alone, independently confirming that these 2 clusters were driven by sample-specific rather than biological structure. These 3 clusters were omitted from subsequent analysis yielding 31 high-quality clusters. Cell-type identification using marker genes indicated 17 neuronal clusters and 14 non-neuronal clusters. The neuronal clusters comprise approximately 62% of all nuclei, with the non-neuronal 37%, the remaining 1% were the discarded clusters 26, 28, and 32. The UMAPs for the RNA, ATAC, and WNN for the neuronal clusters and the non-neuronal clusters are shown in Figs. 2 and 3, respectively. The top 5 marker genes for the neuronal and non-neuronal clusters are in Tables 2 and 3, respectively. Full data are in Table S2 (54).

**Table 1:** 10X Genomics sample output metrics and clustering

	Number of nuclei per cluster by sample								
Batch	2	1	2	1	2	1	2	1	
Clusters	VEH-1	VEH-2	VEH-3	VEH-4	PNA-6	PNA-7	PNA-8	PNA-9	Sum
**0**	738	700	778	739	628	74	731	397	4785
**1**	737	532	763	520	756	70	730	320	4428
**2**	622	639	236	448	863	16	585	261	3670
**3**	498	566	638	493	439	59	506	393	3592
**4**	822	452	461	326	646	32	612	165	3516
**5**	424	439	611	427	446	49	522	324	3242
**6**	379	613	291	494	416	24	461	315	2993
**7**	332	364	546	363	319	37	375	366	2702
**8**	381	450	296	323	426	48	473	272	2669
**9**	350	406	423	319	369	42	317	348	2574
**10**	383	312	365	264	346	33	298	145	2146
**11**	189	265	375	200	209	28	242	203	1711
**12**	128	241	308	255	216	35	327	159	1669
**13**	240	300	140	239	287	4	186	182	1578
**14**	496	125	86	5	416	12	279	4	1423
**15**	112	247	184	182	128	7	104	369	1333
**16**	2	470	186	60	2	1	1	533	1255
**17**	108	121	68	94	110	12	233	8	754
**18**	93	100	93	122	102	21	127	6	664
**19**	102	45	87	110	9	11	41	202	607
**20**	136	79	77	65	115	7	99	27	605
**21**	90	81	95	57	80	10	88	34	535
**22**	88	44	51	46	53	4	37	105	428
**23**	54	71	51	44	56	2	52	32	362
**24**	47	58	60	57	20	6	38	73	359
**25**	26	99	34	34	37	2	34	93	359
**26**	5	2	290	2	0	0	1	3	303
**27**	21	52	66	31	25	4	30	72	301
**28**	3	2	0	0	6	169	1	2	183
**29**	62	11	12	29	8	0	15	34	171
**30**	20	17	22	21	16	0	27	22	145
**31**	20	17	22	21	16	0	27	22	125
**32**	10	4	9	5	6	1	10	7	52
**m33**	6	6	8	5	4	0	6	6	41
**Sum**	7112	7936	7722	6395	7592	823	7609	5491	51 280

**Table 2:** Top 5 neuronal cluster marker genes and percent expressing

#	Postulated Identity	Neuropeptide genes	Classical neurotransmitter genes	Steroid/ neuropeptide receptor genes	Top 5 marker genes
1	GABAergic	*Nxph1* 69%	*Gad2* 92% *Gad1* 77% *Slc32a1* 51%	*Esr1* 32%	*Trpc4* 55% *Grm8* 59%
2	GABAergic Tac1+/Penk+	*Tac1* 68% *Penk* 46% *Cartpt* 46% *Pdyn* 33%	*Gad2* 98% *Gad1* 79% *Slc32a1* 51%	None	*Rarb* 97% *Meis2* 92% *Adamts3* 96% *Foxp2* 83% *Gm10754* 62%
3	GABAergic Sst+	*Sst* 33%	*Gad2* 97% *Gad1* 94% *Slc32a1* 65%	*Pgr* 51%	*Klhl1* 92% *A330008L17Rik* 83% *Chrm2* 86% *Satb1* 81% *Cntnap5c* 64%
4	Mixed GABA/Glut Esr1+ Ar+	*Gal* 28%	*Slc17a6* 45%	*Ar* 53% *Esr1* 51%	*Esr1* 51% *Pde1c* 61% *Prlr* 76% *Ar* 53% *Zim1* 57%
5	Mixed GABA/Glut Nxph1+/Zic1+	*Nxph1* 50%	*Gad2* 87% *Slc17a6* 30%	*Pgr* 40%	*Trpc4* 55% *Gm13629* 63% *Zic1* 60% *St8sia4* 53%
6	Weakly GABAergic Tac1+	*Tac1* 95%	*Gad1os* 19%	*Npy1r* 64%	*Gpr149* 94% *Syt6* 93% *Tac1* 95% *Rreb1* 94% *Htr4* 94%
10	Glutamatergic Adcyap1+	*Adcyap1* 34%	*Slc17a6* 79%	*Nr3c1* 67% *Pgr* 38% *Ar* 32%	*Reln* 68% *Tafa1* 80% *Slc17a6* 79% *Prlr* 64% *Dach1* 67%
12	GABAergic Nts+	*Nts* 36% *Penk* 40% *Crh* 18%	*Gad2* 94% *Gad1* 80% *Slc32a1* 53%	*Ar* 39% *Pgr* 41% *Esr1* 29% *Npy1r* 19% *Npy2r* 18%	*6330411D24Rik* 60% *D030068K23Rik* 76% *Kcnh8* 54% *Sfta3-ps* 70% *Prlr* 69%
13	GABAergic Penk+	*Penk* 59% *Tac1* 49% *Tac2* 21%	*Gad1* 88% *Gad2* 90% *Slc32a1* 52%	None	*Meis2* 100% *6530403H02Rik* 86% *Tshz1* 97% *Lypd1* 82% *Adamts3* 90%
14	GABAergic Avp+/Vip+	*Avp* 66% *Vip* 16%	*Gad1* 75%	*Vipr2* 58%	*Avp* 66% *Dlk1* 82% *Kcnh8* 79% *Cdc14a* 71% *Syt10* 77%
16	Glutamatergic Cck+	*Cck* 72%	*Slc17a7* 98%	*Ar* 62% *Nr3c1* 90% *Nr3c2* 96%	*Tafa1* 95% *Ndst4* 92% *6530403H02Rik* 96% *Sv2b* 100% *Lmo3* 95%
17	Glutamatergic Adcyap1+/Sim1+	*Adcyap1* 56% *Trh* 43%	*Slc17a6* 87%	*Esr1* 43%	*Ebf1* 66% *Sim1* 85% *Angpt1* 54% *Slc17a6* 87% *Vwc2* 76%
18	Glutamatergic Oxt+/Avp+	*Oxt* 63% *Avp* 45% *Sst* 41%	*Slc17a6* 71%	*Nr3c1* 74%	*Sim1* 89% *Fign* 59% *Dach1* 66% *Caprin2* 55% *Angpt1* 54%
23	Cholinergic Chat+/Slc5a7+	*Nxph1* 70% *Agrp* 43%	*Slc5a7* 98% *Chat* 96% *Slc18a3* 41% *Gad2* 91% *Slc17a8* 34%	None	*Slc5a7* 98% *Chat* 96% *Chrm2* 96% *Sv2c* 88% *Prima1* 94%
25	GABAergic Npy+/Sst+	*Sst* 35% *Npy* 31% *Cck* 26%	*Gad1* 94% *Gad2* 94% *Slc32a1* 45%	*Nr3c2* 78%	*Il1rapl2* 61% *Col19a1* 52% *Kcnmb2* 75% *Synpr* 54% *Maf* 67%
31	GABAergic Bnc2+/Ebf1+	*Nxph1* 77%	*Gad2* 97% *Gad1* 89% *Slc32a1* 60%		*Bnc2* 97% *Ebf1* 90% *Plch1* 58% *Ntn1* 86% *Cntnap3* 66%
m33	Glutamatergic Gnrh1+	*Gnrh1* 97% *Pnoc* 42%	*Slc17a6* 73%	*Kiss1r* 47%	*Gnrh1* 97% *Pde11a* 100% *Frem1* 95% *Spag16* 97% *Meis1* 97%

Not all clusters had 5 genes that met the marker gene criteria of *P*_adj_ < .05, log2FC > 0.585, % in cluster >50%, % out cluster <30%.

**Table 3:** Top 5 non-neuronal marker genes and percent expressing

#	Postulated identity	Primary identity genes % expressed	Steroid receptors	Top 5 marker genes
0	Astrocyte Protoplasmic	*Aqp4* 60% *Slc1a3* 97% *Atp1a2* 98% *Slc1a2* 99%	None	*Nhsl1* 93% *Slc6a11* 96% *Gm3764* 95% *Itih3* 82% *Slc39a12* 85%
7	Oligodendrocyte-precursor cells quiescent	*Pdgfra* 92% *Vcan* 92% *Olig1* 67% *Sox10* 62%	None	*Pdgfra* 92% *Vcan* 92% *Gm4876* 88% *Arhgap31* 81% *Cspg4* 70%
8	Oligodendrocytemature	*Mog* 99% *Mobp* 100% *Mag* 96% *Mal* 96% *Tspan2* 99%	None	*St18* 100% *Rnf220* 100% *Mog* 99% *Mal* 96% *Enpp2* 100%
9	Microglia homeostatic	*Cx3cr1* 80% *Tgfbr1* 96% *P2ry12* 86% *Fcrls* 61% *Gpr34* 67%	None	*Tgfbr1* 96% *Apbb1ip* 86% *Inpp5d* 86% *Lrmda* 86% *Ikzf1* 79%
11	Oligodendrocytenew	*Fa2h* 74% *Elovl7* 74% *Mobp* 48% *Mag* 59%	None	*9630013A20Rik* 97% *Bcas1* 99% *Tns3* 98% *Tcf7l2* 95% *Cnksr3* 64%
15	Astrocyte fibrous	*Aqp4* 69% *Slc1a2* 100% *Slc1a3* 99% *Atp1a2* 98%	None	*Mertk* 95% *Prex2* 95% *Gli3* 92% *Nhsl1* 91% *Aqp4* 69%
19	Vascular	*Col3a1 78%* *Dcn. 73%* *Slc6a20a* 80% *Bmp6* 84% *Col1a1* 78%	*Pgrmc1* 80% *Nr3c1* 68%	*Igf2* 91% *Slc7a11* 89% *Cped1* 99% *Ranbp3l* 79% *Slc6a20a* 80%
20	Ependymal cells	*Foxj1* 73% *Ccdc153* 75% *Tmem212* 77% *Mlc1* 84% *Sox9* 92%	*Pgrmc1* 72% *Pgrmc2* 46% *Nr3c1* 65% *Nr3c2* 68%	*Cfap299* 81% *Dnah12* 78% *Tmem212* 77% *Rarres2* 84% *Nudt4* 87%
21	Astrocyte specialized	*Slc1a2* 100% *Slc1a3* 98% *Atp1a2* 99% *Slc4a4* 100% *Slc39a12* 88%	*Esr1* 25% *Pgrmc1* 80% *Ar* 34% *Nr3c1* 82% *Nr3c2* 88%	*Nhsl1* 94% *Plce1* 86% *Ednrb* 86% *Slc39a12* 88% *Gja1* 90%
22	Endothelial cells	*Cldn5* 93% *Ptprb* 94% *Adgrl4* 94% *Slco1a4* 92% *Pecam1* 81%	*Nr3c1* 80% *Pgrmc2* 30%	*Flt1* 99% *Slco1a4* 92% *Rgs5* 67% *Cxcl12* 68% *Ptprb* 94%
24	Microglia border	*Ptprc. 72%* *C1qa 67%* *C1qc 63%* *Runx1 62%*	*Nr3c1* 69%	*Mrc1* 66% *F13a1* 55% *Arhgap15* 68% *Dab2* 70% *Ptprc* 72%
27	Oligodendrocyte-precursor cells proliferating	*Pdgfra* 54% *Vcan* 66%	None	*Pdgfra* 54% *Diaph3* 50% *Vcan* 66% *Arhgap31* 53% *Gm38505* 66%
29	Vascular cells	*Col1a2* 85% *Col12a1* 83% *Col3a1* 65% *Mgp* 89%	*Nr3c1* 68% *Nr3c2* 97%	*Bnc2* 100% *Ptgds* 72% *Adamtsl3* 92% *Mgp* 89% *Fbxl7* 99%
30	Pericytes	*Atp13a5* 94% *Rgs5* 84% *Pdgfrb* 93% *Cald1* 97% *Abcc9* 77%	None	*Atp13a5* 94% *Ebf1* 99% *Rgs5* 84% *Itga1* 98% *Vtn* 91%

Not all clusters had 5 genes that met the marker gene criteria of *P*_adj_ < .05, log2FC > 0.585, % in cluster >50%, % out cluster <30%.

### Marker gene characteristics of neuronal clusters

**Figure 4:**
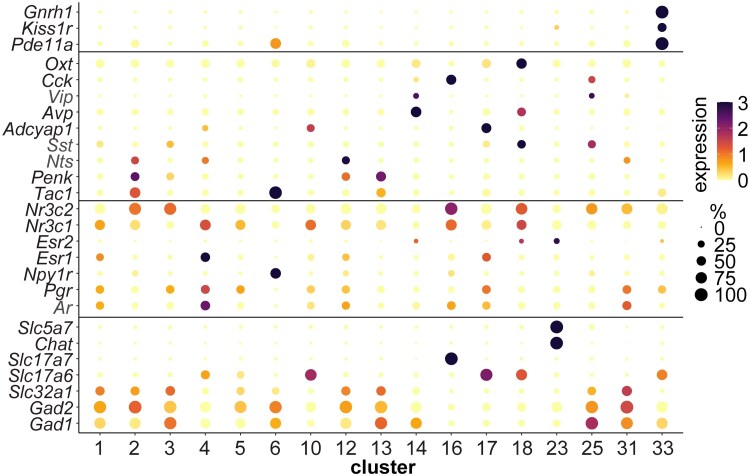
Marker gene expression across neuronal cell populations. Dot plot showing expression of accepted neuronal markers across neuronal clusters. Dot size represents the percentage of nuclei expressing each gene within the cluster, and color intensity indicates the scaled average expression level. GnRH neuron markers (*Gnrh1, Kiss1r, Pde11a)*; neuropeptide markers (*Oxt, Cck, Nrp, Vip, Adcyap1, Tac2, Nts, Penk, Cartpt*); steroid hormone and neuropeptide receptors (*Nr3c2, Esr2, Esr1, Pgr, Ar, Npy1r)*; and neurotransmitter synthesis and transport markers (*Slc17a7, Slc17a6, Slc32a1, Gad2, Gad1, Chat, Slc5a7)*. Expression patterns demonstrate distinct molecular signatures enabling cell type.

Clustering resulted in 17 neuronal clusters; their marker genes are shown in Fig. 4 and Table 2. Of the 17 neuronal clusters, 9 (clusters 1, 2, 3, 6, 12, 13, 14, 25, and 31) were primarily GABAergic and expressed substantial levels of the key genes in gamma aminobutyric acid (GABA) synthesis (*Gad1* and *Gad2,* GAD67 and GAD65, respectively) and vesicular transport (*Slc32a1*, VGAT). Five clusters (10, 16, 17, 18, and 33) were primarily glutamatergic and expressed the vesicular glutamate cotransporters *Slc17a7* and *Slc17a6* (VGUT1 and VGUT2), including the GnRH neurons. Clusters 4 and 5 were mixed GABA/glutamate populations. Cluster 23 was the sole cholinergic population, expressing the choline transporter *Slc5a7* and the acetylcholine synthetic enzyme *Chat* (choline acetyltransferase). Other neuronal clusters were identified by neuropeptides and/or transcription factors including: magnocellular neurons (cluster 18) expressing both oxytocin (*Oxt*) and vasopressin (*Avp*); neurons likely involved in energy regulation expressing cholecystokinin (*Cck*, clusters 16 and 25), *Sim1* (Cluster 17) or *Bnc2* (cluster 31); a population (cluster 14) involved in osmoregulation and/or circadian rhythms expressing vasopressin (*Avp*) and vasoactive peptide (*Vip*); populations (clusters 12 and 13) involved in opioid signaling expressing proenkephalin (*Penk*), and tachykinin populations expressing *Tac1* (clusters 2 and 6). Notably, the only neuronal population that expresses *Esr1* and *Ar* steroid receptors to the level required for identification as marker genes was the mixed GABA/glutamate cluster 4. Other clusters expressed these genes but at a lower percentage and/or less exclusive manner, including 1, 3, 10, 12, 16, 17, and 23.

### Strength of *Gnrh1* expression allows for manual clustering

**Figure 5:**
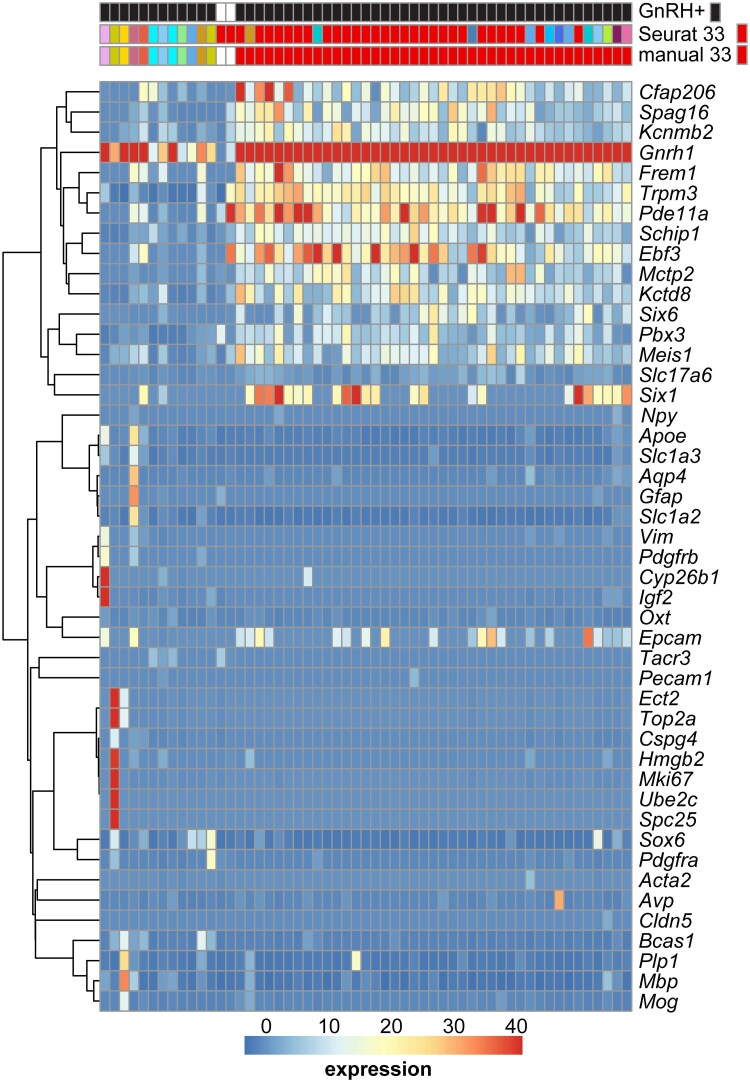
Pearson coefficient heat map of gene expression in all nuclei in Seurat cluster 33 and the additional 24 nuclei that had ≥5 *Gnrh1* “reads.” Top row, *Gnrh1* expression (black). Second row, color-coded cluster assigned by Seurat (see Figs. 2 and 3; cluster 33 in red). Third row, manual reassignment of nuclei to m33 (red) and removal of 2 non-*Gnrh1*-expressing nuclei from Seurat cluster 33 (white). Below, expression heat map of selected genes. Dendrogram of associations is on the left.

GnRH neurons are a rare cell type (∼800-1000 in mice) (67, 68) but are distinguished by strong and quite cell-specific expression of *Gnrh1*. This led us to test if manual clustering would increase the yield of GnRH neurons for single-cell analysis. Nuclei with ≥5 raw *Gnrh1* “reads” were selected and the 2000 most variable genes were compared to the Seurat cluster 33 (s33)-determined GnRH neurons. An additional 24 nuclei with *Gnrh1* > 5 (total = 53) were identified. They were compared to the Seurat-33 cluster in a heatmap after hierarchical clustering. Figure 5 shows a representative portion of the heatmap used for this characterization, for the full heatmap of the top 3000 most variable genes see Fig. S2 (54). Twelve of the high *Gnrh1*-expressing nuclei outside of cluster 33 had the signature of the cluster they were originally sorted into (Fig. 5, top left). For example, the nucleus in column 1 on the far left was in cluster 19, vascular cells; despite 6 raw *Gnrh1* counts, it still expresses genes such as *Vim*, *Pdgfrb*, *Cyp26b1,* and *Igf2* that are prevalent in mesenchymal cells such as vascular smooth muscle, pericytes, and endothelial cells. This cell further lacks expression of the identified GnRH neuron markers including *Cfap206*, *Spag16*, *Kcnmb2*, *Frem1*, *Pde11a*, *Ebf3*, *Mctp2*, *Six6*, and *Meis1* (discussed below). These high *Gnrh1*-expressing non-GnRH cells likely represent technical artifacts such as ambient RNA contamination remained in their assigned cluster. In contrast, the other 12 nuclei originally outside of cluster 33 that had high *Gnrh1* expression had transcript signature patterns resembling those in cluster 33 (upper right quadrant of Fig. 5) and thus were included in manual cluster 33 (m33). Two of the nuclei Seurat placed in cluster 33 did not express *Gnrh1* and their signature diverged from the other nuclei in that cluster suggesting they were also algorithmically misclassified. These were removed from m33 and not analyzed further. Note, although scDblFinder flagged 10 of the 12 reclaimed GnRH nuclei as doublets, further analysis reveals this is likely a computational mischaracterization. The SCTransform residual heatmaps (Fig. 5 and Figs. S2 and S3 (54)) show that each rescued nucleus robustly expresses the GnRH program with few of the marker genes of its original cluster. As true physical doublets would display a hybrid, dual-lineage signature, these clean signatures suggest they are highly active, single GnRH nuclei. For complete transparency, all analyses for both the algorithmic and manual GnRH clusters were conducted in parallel. The main manuscript displays the results for the manual cluster (m33), whereas the corresponding data for the Seurat algorithmic cluster (s33) are in the supplementary files. Marker genes for both the m33 and s33 are in Table S2 (54).

m33 contains 41 nuclei (s33 minus 2 false positives plus 12 validated additions), providing a detailed definition of GnRH neurons based on both *Gnrh1* expression and overall transcriptional identity. Key GnRH neuron marker genes (*Cfap206*, *Spag16*, *Kcnmb2*, *Gnrh1*, *Frem1*, *Pde11a*, *Ebf3*, *Mctp2*, *Six6*, *Meis1*) all meet strict marker criteria (*P*_adj_ < .05, log2FC > 0.585, percent in cluster ≥ 50%, percent outside cluster <30%). Additional marker genes including *Trpm3*, *Schip1*, *Kctd8*, and *Pbx3* showed significant enrichment in GnRH neurons (*P*_adj_ < .05, log2FC ≥ 0.585) but with broader expression across other cell types (percent outside cluster >30%). *Epcam* (epithelial cell adhesion molecule), previously identified as enriched in GnRH neurons by TRAP (39, 69), was expressed in approximately 45% of GnRH neurons but only 8% of all nuclei, confirming its enrichment in this population despite variable expression.

The manual cluster (m33, 41 nuclei) and algorithmic cluster (s33, 31 nuclei) share 26 marker genes (40% of the combined set). The 35 markers unique to m33 are also present in s33 with similar fold-change but had failed to achieve marker status due to either reduced statistical power (32/35) or <0.5% in cluster (3/35). This pattern of very similar log2FC and improved statistical resolution are what is expected when additional true members of a rare cell type are correctly assigned to their cluster. The 4 markers (*Gm27151*, *Dcdc2a*, *Kiss1r,* and *Vav2*) that are unique to s33 represent threshold boundary effects rather than biological differences. For example, *Kiss1r*, a well-established GnRH neuron marker expressed in approximately half of these GnRH neurons, narrowly misses the strict averaged marker threshold in m33 (log2FC = 0.548, percent in cluster = 0.47) compared to s33 (log2FC = 0.60, percent in cluster = 0.54). The same is true for *Dcdc2a* and *Gm27151*, only *Vav2* loses significance in m33 vs s33 (*P*_adj_ = .281 and .001, respectively). Therefore, the manual cluster is not an artifact of overly liberal inclusion, but evidence that the added nuclei are authentic GnRH neurons.

### Marker gene characteristics of non-neuronal clusters

**Figure 6:**
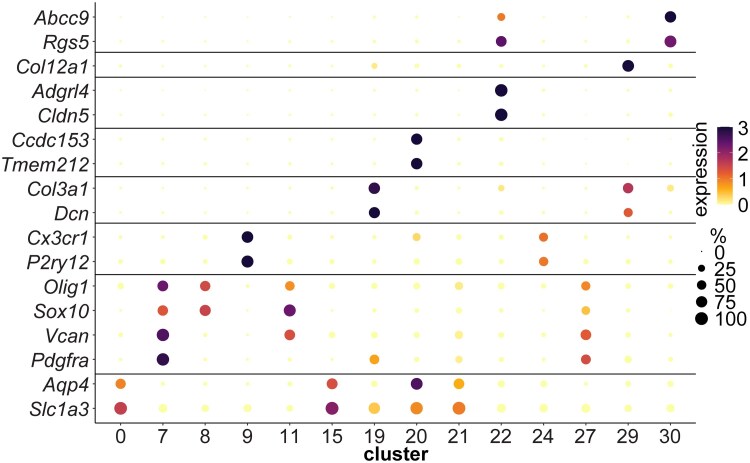
Marker gene expression across non-neuronal cell populations. Dot plot showing expression of canonical cell-type markers across non-neuronal clusters identified at resolution 0.25. Dot size represents the percentage of nuclei expressing each gene within the cluster, and color intensity indicates the scaled average expression level. Markers include astrocyte markers (*Aqp4*, *Slc1a3*); oligodendrocyte lineage markers (OPC and mature oligodendrocytes, *Olig1, Sox10, Vcan, Pdgfra)*; microglial markers (*Cx3cr1*, *P2ry12*); ependymal cell markers (*Ccdc153*, *Tmem212*); and vascular markers including general markers (*Col3a1*, *Dcn*, *Col12a1*); endothelial markers (*Cldn5*, *Adgrl4*), and pericyte markers (*Abcc9*, *Rgs5*). Expression patterns demonstrate distinct molecular signatures enabling classification of non-neuronal hypothalamic populations.

Clustering resulted in 14 non-neuronal clusters; their marker genes are shown in Fig. 6, Table 3, and Table S2 (54). Three astrocyte subtypes (clusters 0, 15, and 21) expressed the pan-astrocyte genes *Aqp4, Slc1a2, Slc1a3, and Gja1*. Clusters 0 and 21 are protoplasmic-type astrocytes that express the GABA transporters *Slc6a11* and *Itih3* with Cluster 21 also expressing *Plce1* and *Spon1*, which are involved in synaptogenesis (70). Cluster 15 is a fibrous astrocyte subtype with the highest expression of the glutamate transporters *Slc1a2* and *Slc1a3* and the metabotropic glutamate receptor *Grm3*, suggesting specialized glutamate handling.

Clusters 7, 8, and 11 were identified as oligodendrocyte lineage by expression of *Olig1* and *Sox10* transcription factors (Table 3). Clusters 7 and 27 are oligodendrocyte precursor cells (OPCs) expressing *Pdgfra* and *Vcan*. Cluster 27 additionally expresses proliferation-associated markers *Diaph3* and *Ezh2*, marking it as proliferating OPCs (Table S2 (54)). Cluster 11 represents newly formed oligodendrocytes that retain persistent *Vcan* expression alongside the premyelinating markers *9630013A20Rik* and *Bcas1*. Cluster 8 represents mature myelinating oligodendrocytes expressing *Mog* and *Mag*.

Cluster 20 comprises ependymal cells lining the third ventricle, characterized by expression of genes critical for motile cilia including *Foxj1*, *Ccdc153*, *Tmem212*, and dynein heavy chain genes *Dnah12* and *Dnah6* that power ciliary beating. These cells also express *Ocln*, *Aqp4*, and *Mlc1*, consistent with their roles in barrier function. The transcriptional specialization of this population is reflected in over 500 marker genes, the most of any cluster in this dataset (Table 3, Table S2 (54)).

Two immune cell populations were identified. Cluster 9 is homeostatic microglia expressing *Tgfbr1*, the fractalkine receptor *Cx3cr1*, and purinergic receptor *P2ry12*. Cluster 24 is border-associated macrophages distinguished from homeostatic microglia by expression of the mannose receptor *Mrc1*, *F13a1*, and *Arhgap15* (Table 3), with immune identity confirmed by the pan-leukocyte marker *Ptprc*, complement genes *C1qa* and *C1qc,* and the myeloid transcription factor *Runx1*.

The remaining 4 clusters are vascular and perivascular cell types. Cluster 22 is endothelial cells expressing barrier and transport genes including *Cldn5, Flt1, Pecam1, Slco1a4*, and *Slco1c1*. Cluster 30 is pericytes, mural cells wrapping brain capillaries, expressing contractile and signaling genes *Pdgfrb, Rgs5, Cald1*, and *Atp13a5*. Clusters 19 and 29 are fibroblast-like cells expressing extracellular matrix and lipid signaling genes *Col3a1, Dcn*, and *Ptgds*, consistent with leptomeningeal and perivascular fibroblast populations.

### Gene enrichment varies between VEH and PNA

**Figure 7:**
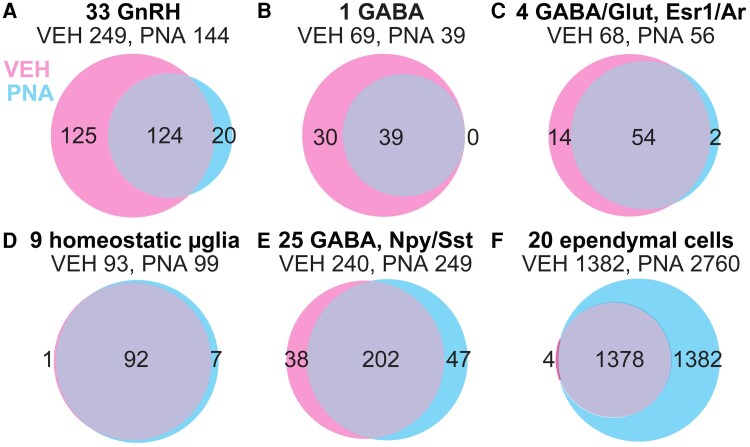
Proportional Venn diagrams of genes enriched in VEH (magenta) and PNA (cyan) snRNAseq data. Six example clusters are shown that range from being more specifically determined in VEH at the upper left to more specifically determined in PNA on the lower right. Label at the top is cluster number and main cell type attributed to that cluster.

Marker gene identification above required both selectivity and exclusivity within a particular cluster. For analysis of gene enrichment, we required significant enrichment within cluster vs outside cluster (*P*_adj_ < .05, log2FC ≥ 0.585), but not exclusivity. This analysis revealed differences between VEH and PNA samples. VEH samples generally had a greater number of treatment-specific enriched genes per cluster than PNA samples (Fig. 7). Eight clusters exhibited more enriched genes in VEH than PNA samples, while only one cluster (ependymal cells) had more in PNA. The most pronounced asymmetries occurred in GnRH neurons (m33: 249 VEH vs 144 PNA-enriched genes, 2.7-fold difference) and GABAergic neurons (cluster 1: 69 VEH vs 39 PNA-enriched genes, 1.8-fold difference). Ependymal cells (cluster 20) are a notable exception, with PNA-treated samples exhibiting nearly twice as many enriched genes as VEH (2760 vs 1382). This PNA-associated expansion in ependymal cells was not driven by specific functional categories but rather reflected broad, low-level activation of diverse cellular processes (median log2FC ∼0.70), suggesting reduced transcriptional specialization rather than activation of discrete gene programs.

### Results of differential gene analyses

To assess if prenatal androgen exposure altered hypothalamic transcription at the tissue level, counts across all nuclei were aggregated within each sample to create pseudobulk sample-level profiles and principal component analysis performed using the 2000 most variable genes. This analysis revealed that sample-to-sample variation was driven primarily by differences in hypothalamic dissection (PC1: 56% variance) rather than treatment effects, as PNA and VEH samples overlapped (Fig. S4 (54)). Gene set enrichment analysis of PC1's loadings indicate enrichment for GO:0035249 synaptic transmission, glutamatergic (NES 2.01, false discovery rate [FDR] = 0.0116), and 39 of the top 40 genes with the most positive loadings for PC1 are enriched in cluster 16 (glutamatergic, CCK). Consistent with this, VEH-2 and PNA-9, the samples most positively displaced along PC1, are overrepresented in cluster 16, contributing 37% and 42% of the total nuclei, respectively (Table 1). To control for cellular composition effects and increase statistical sensitivity for detecting treatment-specific changes, we focused subsequent differential expression analyses at the cluster level.

Pseudobulk differential expression analysis at the cluster level revealed no individual transcripts reaching statistical significance after multiple testing correction (FDR < 0.05), despite nominal differences (*P* < .05) across multiple genes in all clusters (Table S3 (54)). As the dataset is limited, we examined differential expression at relaxed criteria (|log2FC| > 0.58, FDR < 0.25) to explore transcriptional trends below our primary significance threshold. This analysis identified a small number of nominally differentially expressed genes across 6 clusters (Table 4). In GnRH neurons (m33), *Edil3* was nominally upregulated in PNA relative to VEH animals. In cluster 21, a potential perisynaptic astrocyte population marked by high *Egfr* and *Slc7a11* expression, several genes showed nominally lower expression in PNA relative to VEH animals, including *Irf2bp2* and *Nr6a1*, while *Phgdh* was nominally elevated in PNA.

**Table 4:** Differentially expressed genes FDR < 0.25

Cluster/gene	log2FC	*P* value	FDR	Direction
**6 GABA, Tac1**				
* Hcn1*	0.96	6.21E−06	0.0702	PNA↑
* Gm47469*	1.70	6.61E−06	0.2488	PNA↑
* Gm15614*	0.90	4.68E−05	0.2488	PNA↑
**13 GABA, Penk**				
* Shisa6*	−0.74	9.85E−06	0.1173	VEH↑
**17 Glutamate, Adcyap1**				
* Gm45904*	1.31	1.92E−05	0.2020	PNA↑
**18 Glutamate, Oxt/Avp**				
* Pbx3*	−0.74	1.42E−05	0.1422	VEH↑
**21 Astrocytes, specialized**				
* Hcn1*	−0.62	6.44E−05	0.1193	VEH↑
* Irf2bp2*	−1.16	3.01E−05	0.1193	VEH↑
* Gm13481*	−1.36	7.88E−05	0.1193	VEH↑
* Phgdh*	1.16	3.02E−05	0.1193	PNA↑
* Galnt17*	−0.65	4.25E−05	0.1193	VEH↑
* Nr6a1*	−0.72	9.04E−05	0.1198	VEH↑
**33 GnRH**				
* Edil3*	2.68	4.84E−05	0.0798	PNA↑

### GSEA analysis

**Figure 8:**
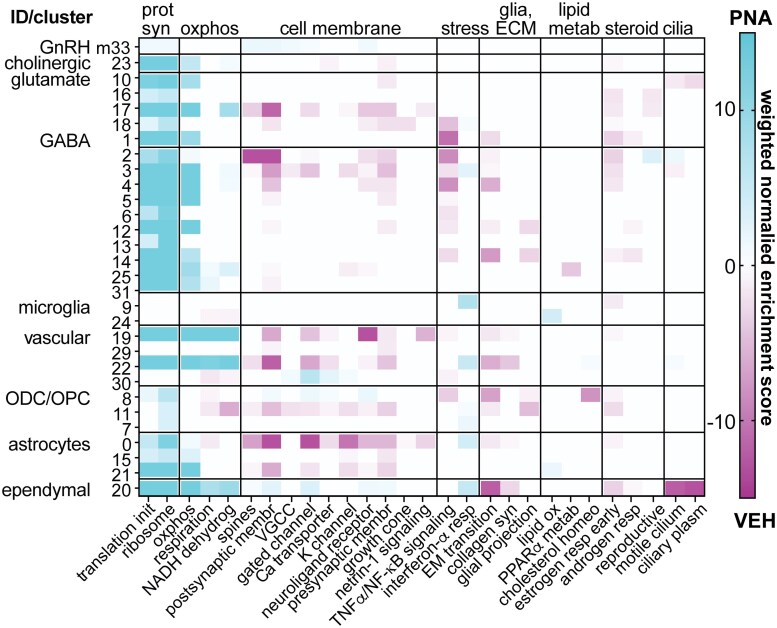
Gene set enrichment analysis of pseudobulk differential expression. For each cluster, results from the pseudobulk differential expression analysis were ranked by log2 fold change (+PNA to −VEH) without pre-filtering by significance. Ranked gene lists were tested against 6 curated gene set databases retrieved via the MSigDB package for *Mus musculus*: GO Biological Process, GO Molecular Function, GO Cellular Component, KEGG, Reactome, and MSigDB Hallmark gene sets. Cluster 27 had no enrichment in either direction and is omitted. Data are presented as a weighted NES score; signed NES×−log10(*P*_adj_ ). Positive weighted NES (Aqua) is in the direction of PNA. Negative weighted NES (purple) is in the direction of VEH. For ease of viewing scores have been capped at −10 and 10. For all values (NES, *P*_adj_ , leadingEdge genes, etc.) see Table S4 (54). Abbreviations: dehydrog, dehydrogenation; ECM, extracellular matrix; EM transition, epithelial mesenchymal transition; homeo, homeostasis; init, initiation; LTP, long-term potentiation; membr, membrane; metab, metabolism; ox, oxidation; oxphos, oxidative phosphorylation; prot, protein; resp, response; syn, synthesis; VGCC, voltage-gated Ca channel.

To identify potential pathway-level signals not detectable at the level of individual genes, we performed exploratory GSEA (Fig. 8). Results should be interpreted as hypothesis-generating, particularly for the sample sizes involved. GSEA revealed several putative pathway-level responses to PNA treatment. Protein synthesis (translation and ribosome) and oxidative phosphorylation were enriched in PNA samples spanning neuronal, glial, and vascular cell types. PNA samples were broadly, but not totally, de-enriched for steroid-responsive gene sets. These gene sets were enriched in VEH samples across 8 clusters including GABAergic (clusters 1, 2, 3, 4), glutamatergic (clusters 16, 17), oligodendrocyte (cluster 11), and ependymal (cluster 20) populations. Androgen response gene sets were depleted in PNA samples from clusters 1, 12, and 14. There were also treatment-associated shifts in stress response gene sets. Homeostatic TNFα/NF-κB signaling was enriched exclusively in VEH samples in neuronal clusters and was de-enriched in PNA samples in some clusters. Finally, the only significant findings in GnRH neurons were enrichment in PNA samples of GO cellular component terms reflecting genes localized to postsynaptic membranes, dendritic spines, and protein synthesis. This is consistent with remodeling of synaptic architecture rather than broad transcriptional reprograming. Full data available in Table S4 (54).

### Chromatin accessibility of marker genes

**Figure 9:**
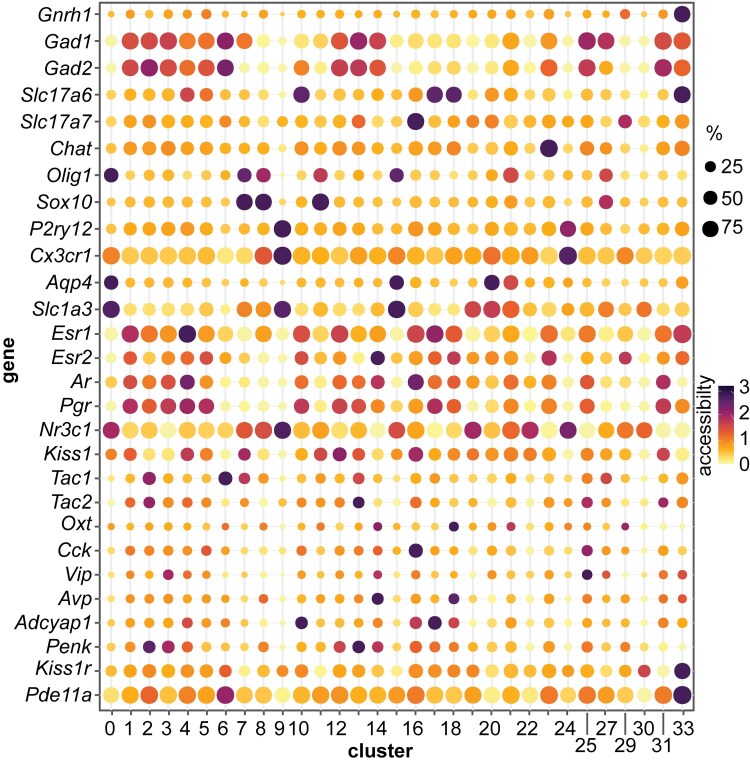
GeneActivity dotplots of chromatin accessibility at selected gene loci across all clusters. GeneActivity scores were computed by Signac GeneActivity() function, which quantifies chromatin accessibility at gene loci by counting ATAC-seq fragments overlapping each gene body (from transcription start site to transcription end site), normalized by gene length. This produces a per-nucleus accessibility score that is then averaged across nuclei within each cluster. Dot size reflects the percentage of nuclei within a cluster with detectable accessibility (score > 0) and color intensity reflects the average GeneActivity score across all nuclei in the cluster.

Chromatin accessibility at marker gene loci across clusters is shown in Fig. 9. Chromatin accessibility at cluster-defining gene loci broadly recapitulates the transcriptional identities established by snRNA-seq clustering. The *Gnrh1* locus is accessible primarily in m33. All GABAergic neuronal clusters show preferential accessibility at *Gad1* and *Gad2*, glutamatergic clusters at *Slc17a6* and/or *Slc17a7*, oligodendrocyte lineage clusters at *Olig1* and *Sox10*, microglial clusters at *P2ry12*, and astrocyte clusters at *Aqp4* and *Slc1a3*, consistent with the transcriptional identities assigned by marker gene analysis.

### Differential analysis of chromatin accessibility by prenatal treatment

As with GEX data, *P*-value distributions across all but m33 were right-skewed or hill-shaped, consistent with an underpowered pseudobulk design (Scenario F; (71)) reflecting the limitations of pseudobulk ATAC-seq testing with 3 to 5 biological replicates per group. Despite this, surviving hits at FDR < 0.05 are likely to represent genuine biological signals, as conservative underpowering increases Type II errors (false negatives) rather than Type I errors (false positives). An interesting exception was m33, which displayed a uniform *P*-value distribution consistent with the expected null, likely reflecting the small number of nuclei in this cluster triggering minimum count filters that restrict testing to robustly expressed peaks.

**Figure 10:**
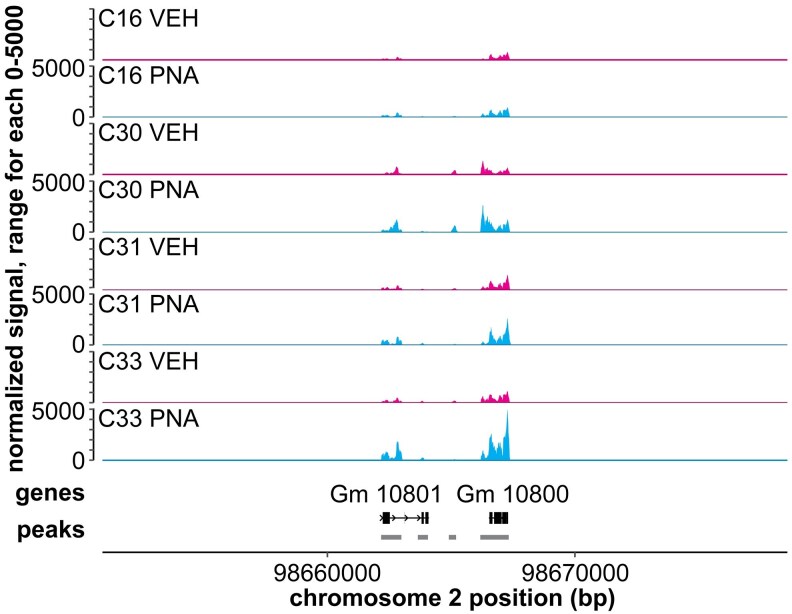
Coverage plot of chromatin accessibility at chr2:98,662,1718662171-98,662,9888662988 illustrating the increased accessibility of this locus in multiple clusters in PNA-treated mice.

Across all clusters, 13 unique peaks passed FDR < 0.05 (Table 5, Table S5 (54)) and were distributed across multiple cell types and chromosomes, with a subset in multiple clusters. The most significant differentially accessible region (Fig. 10) was a peak on chromosome 2 (mm10: chr2:98,662,1718662171-98,662,9888662988) enriched in PNA animals (FDR < 0.05) in 3 independent clusters (clusters 33, 31, and 16) and nominal significance in a fourth (cluster 30). This intergenic region, proximal to the predicted gene *Gm10801*, has no characterized gene annotation and represents the strongest and most replicated chromatin accessibility signal in the dataset. A second PNA-enriched peak in GnRH neurons (m33) was identified at an intergenic locus on chromosome 9 (mm10: chr9:35,305,1205305120-35,305,5635305563), also with no proximate RefSeq gene annotation. In VEH mice, a recurring locus on the X chromosome (mm10: chrX:106,186,28306186283-106,188,13606188136) was enriched in 3 glial clusters (astrocytes, cluster 0; homeostatic microglia, cluster 9; newly formed oligodendrocytes, cluster 11) and nominal significance in 5 additional non-neuronal clusters. Genomic annotation revealed this peak maps to the promoter region of *Pgk1* (phosphoglycerate kinase 1), a housekeeping glycolytic enzyme.

**Table 5:** Differential chromatin accessibility peaks between VEH and PNA samples

Peak (mm10)	Gene	Annotation	Cluster(s)	Change	log2FC	FDR
**PNA-enriched peaks, FDR < 0.05**						
chr2:98662171-98662988	Intergenic (*Gm10801*)	Intergenic candidate cis-regulatory elements (cCRE); repeat-flanked; no characterized gene body	33 GnRH 16 Glut/CCK 31 GABA/Bnc2	PNA↑	0.68-1.80	6.8E−06-1.7−02
chr2:98666179-98667327	Intergenic (*Gm10800*)	Intergenic cCRE; same locus as above	30 Pericytes	PNA↑	1.00	3.6E−05
chr9:35305120-35305563	Intergenic	Intergenic enhancer-marked region; no RefSeq gene	33 GnRH	PNA↑	1.73	3.2E−04
chr2:162086626-162088000	*Ptprt*	Intronic cCRE (enhD; receptor tyrosine phosphatase; synaptic signaling/STAT3)	13 GABA/Penk	PNA↑	2.18	2.1E−02
chr13:31179022-31179991	Intergenic	Distal enhancer cluster	9 Microglia, homeostatic	PNA↑	1.79	4.5E−02
**VEH-enriched peaks, FDR < 0.05**						
chrX:106186283-106188136	*Pgk1*	Promoter/TSS; glycolytic housekeeping	9 Microglia 0 Astrocyte protoplasmic 11 oligodendrocyte, new, *20 Ependymal, *P*_adj_ = .13	VEH↑	0.61-0.73	4.0E−05-2.9E−03
chr5:135777665-135779310	*Mdh2*	Promoter/enhP (ENCODE cCRE); TCA cycle/mitochondrial metabolism; broad expression	31 GABA/Bnc2	VEH↑	1.24	1.9E−02
**Exploratory peaks (0.05** **≤** **FDR < 0.25**						
chr9:56795360-56797251	*Lingo1*	Promoter cCRE cluster (enhP + prom + CTCF); inhibitor of oligodendrocyte differentiation/myelination	8 Oligodendrocytes, mature	PNA↑	0.71	0.107
chr18:67342867-67344295	*Cidea*	Promoter cCRE cluster (2× prom + enhP); lipid droplet/lipid metabolism gene	11 Oligodendrocyte, new	PNA↑	1.22	0.233
**Uncharacterized peaks, FDR < 0.05**						
chr15:75085437-75087052	*Gm28502*	Predicted gene; dense RepeatMasker; no ENCODE cCREs	33 GnRH neurons 16 Glut/CCK	PNA↑	0.74-2.81	9.5E−04-3.5E−03
chr2:181916748-181919223	Intergenic	Repeat-rich, non-genic, no cCREs	16 Glut/CCK	PNA↑	1.05	5.9E−05
chr11:109011535-109012086	Intergenic	Repeat-rich, non-genic, no cCREs	16 Glut/CCK	PNA↑	0.66	4.9E−03
chr13:6498396-6499136	Intergenic	Repeat-rich, non-genic, no cCREs	6 GABA/Tac1	VEH↑	1.05	0.052

Among the remaining FDR-significant peaks, cluster 13 (GABAergic/Penk neurons) harbored a PNA-enriched peak within an intronic region of *Ptprt* (mm10: chr2:162,086,62662086626-162,088,00062088000; FDR = 0.021). The *Ptprt* gene (protein tyrosine phosphatase receptor type T) encodes a type IIB receptor-type protein tyrosine phosphatase that plays a critical role in the regulation of synaptic formation, dendritic arborization, and neuronal development (72). Cluster 9 (homeostatic microglia) showed a PNA-enriched intergenic peak on chromosome 13 (mm10: chr13:31,179,0221179022-31,179,9911179991) with an ENCODE candidate cis-regulatory element. Cluster 31 (GABAergic/Bnc2 neurons) contained a VEH-enriched peak at the *Mdh2* promoter (mm10: chr5:135,777,66535777665-135,779,31035779310), consistent with higher oxidative metabolic activity in VEH animals. Additionally, 2 peaks on chromosome 15 within a wide intergenic region reached FDR < 0.05 in clusters 33 and 16; the genomic context of this locus has not been characterized.

Peak-to-gene linkage analysis was performed to identify correlations between differential chromatin accessibility and nearby GEX, but no formal peak-to-gene links could be established for most FDR-significant loci identified. This likely reflects a known limitation of correlation-based linkage methods, which require sufficient cell-to-cell variation in both accessibility and expression to compute meaningful correlations (50). Constitutively expressed or broadly accessible loci, including housekeeping genes such as *Pgk1*, lack within-cluster variance, precluding linkage regardless of differential accessibility between treatments. Functional validation will be needed to demonstrate if these differentially accessible peaks can be interpreted as epigenetic marks of potential regulatory relevance.

## Discussion

Reproductive neuroendocrine function is disrupted in women with hyperandrogenemic PCOS and girls with hyperandrogenemia. Epigenetic changes occur in women with PCOS and in PNA animal models in several non-neural tissues (73-78). While progress has been made in understanding how the physiology of preoptic area cells, including GnRH neurons, is affected in PNA mice, a missing gap is a more granular understanding of changes in chromatin structure and GEX. Single-nucleus RNAseq and ATACseq were combined in preoptic samples from prepubertal VEH and PNA mice. Prepubertal mice were chosen to identify changes that might occur early in phenotypic development as these may provide more effective targets for eventual therapeutic approaches.

For GnRH neurons, our work both supports prior characterizations of an enriched population (39, 69) or single-cell-analysis level (56, 57, 79, 80), and provides the first analysis of chromosomal accessibility. *Gnrh1*-expressing nuclei of the POA clustered with the cholinergic neuron population until application of the 25PC-0.25 resolution analysis combination, suggesting that despite differences in neurotransmitter identity, these cell types share several transcriptional features. Interestingly, extrahypothalamic GnRH-expressing cells also express choline acetyltransferase (81). The strong expression of *Gnrh1* allowed us to refine the Seurat-generated cluster 33 by manually adding nuclei from other clusters that had both high *Gnrh1* reads and a similar pattern of GEX. Manually adding 12 nuclei increased the population of GnRH-expressing nuclei analyzed by ∼30%. The manual cluster is more inclusive, with 61 vs 30 marker genes. The number of high-quality clusters, 31, is greater than typically observed in single-cell analyses, but is consistent with other studies of the hypothalamus/POA and with the high diversity of cell types contained in this relatively small region (57, 82).

GnRH neuron marker genes from m33 were compared with a PND21 TRAP-seq dataset (39) and the HypoMap atlas (57) (Fig. S5 (54)). Eight genes were enriched across all 3 methods establishing a robust GnRH neuron GEX signature. An additional 26 genes were detected by TRAP-seq and HypoMap but not m33, including the established GnRH markers *Tstd1, Rab25, Cldn9, Wfdc1, Fam92b,* and *Smim2* (39, 56, 57, 69). These differences could be biological, arising from different ages and/or treatments. For example, 2 genes exclusive to HypoMap (*Cnksr1*, *Kcng4*) were enriched in adults in the TRAP dataset but not adolescents, and 2 genes (*Capg*, *Gdnf*) were enriched only in VEH pups. More commonly, however, these differences likely reflect single-cell gene dropout in m33 from reduced depth of sequencing in single-cell analyses. Fifteen genes were common to TRAP-seq and m33, including GnRH neuron-critical transcription factors *Six1* and *Six6* (83-85). Five genes were detected by both single-cell methods but not TRAP-seq, perhaps because these transcripts exhibit low ribosome occupancy. *Meis1* and its cofactor *Pbx3* (enriched in m33) are transcription factors critical for GnRH neuron differentiation in the olfactory epithelium (86) and for *Gnrh1* expression in GnRH-derived GT1-7 cells (87). *Pde11a* is a dual-specificity phosphodiesterase that degrades both cAMP and cGMP. In GT1-7 cells, overexpression of exogenous cAMP phosphodiesterase reduced GnRH secretion (88); *Pde11a* may be the endogenous mediator of this regulation. The roles of *Cngb3*, *Frem1,* and *Gm49857* in the GnRH neuron are more speculative and, like many of the genes shared across all these datasets, reflect the GnRH neuron's olfactory placode origin.

To probe changes in GEX in the midventral POA with PNA treatment, 3 levels of analysis were performed: gene enrichment, differential GEX, and physiological pathways via GSEA. Examining gene enrichment allowed relaxation of the exclusivity criteria mandated for marker genes. Most clusters had similar enrichment between VEH and PNA samples, but 8 clusters had more enriched genes in VEH samples, including GnRH neurons, whereas only the ependymal cell cluster 20 had more enriched genes in PNA samples. These patterns suggest PNA treatment may reduce transcriptional distinctiveness in certain cell types. Differential expression of individual genes was not detected in any cluster using FDR <0.05. Relaxing FDR to <0.25 revealed minimal differentially expressed genes in 5 clusters, including nominal upregulation in m33 of *Edil3,* which encodes an integrin-binding matricellular protein that can reduce dendritic spine density in primary hippocampal neurons (89). Its upregulation in PNA GnRH neurons may contribute to PNA-induced alterations in spine density in these cells (33, 90, 91).

In contrast to somewhat unexceptional changes at the single-gene level, analysis of functional gene sets generated several interesting hypotheses regarding PNA-induced changes in different clusters. Most clusters spanning neural, glial, and vascular cell types exhibited 2 broad metabolic changes. First, protein synthesis was enriched in PNA samples. This was also observed in TRAP-seq studies of the GnRH neuron translatome but was not emphasized as TRAP employs overexpression of a tagged ribosomal subunit. This caveat was mitigated with single-nuclear approaches, indicating this upregulation may reflect genuine biology. Second, oxidative phosphorylation genes were enriched in PNA samples. These findings can be reconciled with the TRAP-seq data through differences in analysis methodology. The original TRAP analysis compared GnRH neurons to total preoptic input using over-representation analysis of directionally separated gene lists; this precluded direct comparison of complete pathway activity between treatment groups. Reanalysis of the direct comparison of PNA and VEH TRAP-seq GnRH neuron fractions using GSEA revealed significant oxidative phosphorylation depletion in adult PNA GnRH neurons (NES = −1.49, *P*_adj_ = .031) that was not yet present at PND21 (NES = 0.87, *P*_adj_ = .93), consistent with a progressive developmental metabolic change in GnRH neurons (Table S6 (54)) (39). The present finding of broad PNA-associated upregulation of oxidative phosphorylation across preoptic populations likely explains the finding of a relative reduction in oxidative phosphorylation enrichment in PNA GnRH neurons in the original TRAP analysis. The breadth of this PNA-associated upregulation and the small number of GnRH neurons made this pathway appear reduced in GnRH neurons. These changes in protein synthesis and respiration point to a possible broad upregulation of metabolic activity across the preoptic area in PNA mice.

Additional specialized pathways were also disrupted in PNA samples. First, there was a relative depletion in PNA samples of both estrogen and androgen-responsive gene sets across multiple neural and non-neuronal clusters. Reduced efficacy of steroid feedback has been reported in both peripubertal girls and adult women with PCOS, as well as in PNA animal models (92-95); the present data suggest shifts in steroid-responsive pathways may contribute to this near puberty. Second, there were PNA-associated shifts in stress response gene sets. Homeostatic TNFα/NF-κB signaling was enriched exclusively in some VEH clusters, both neuronal and non-neuronal. Interferon-α response was, in contrast, enriched in PNA glia. Together these results may indicate PNA induces a shift to an active inflammatory state in some cell types. Third, in GnRH neurons, there was enrichment in PNA samples of GO cellular component terms reflecting genes localized to postsynaptic membranes and dendritic spines. This is consistent with remodeling of synaptic architecture, for which there is both physiologic and anatomic evidence in this model (27, 33, 96), rather than broad transcriptional reprograming. While it is important to bear in mind that GSEA is primarily a hypothesis-generating tool, these hypotheses point to potentially productive areas for future research.

The present work provides the first analysis of GnRH neuron chromatin accessibility. *Gnrh1* locus accessibility was greatest in cluster 33 (GnRH neurons), with substantially lower and less consistent signal across other clusters, consistent with cell-type-associated chromatin accessibility at this locus. This serves as a within-study validation of the manual cluster approach. m33 also shows the medium to high accessibility for the 8 marker genes identified by all 3 methodologies (Multiome, *Gnrh1* TRAP-seq, and Hypomap; Fig. S4 (54)), suggesting epigenetic mechanisms play a role in marker gene regulation in this cluster. Of note, chromatin accessibility is highest in m33 for *Kiss1r,* which did not meet the strict criteria for being a marker gene in the present study but encodes a protein vitally important for the function of GnRH neurons (97-100).

Steroid hormones are an essential component of both typical hypothalamic-pituitary-gonadal axis function and pathophysiology in women with PCOS (101) and PNA animal models (6). In the present work, the steroid receptor genes varied in their chromatin accessibility. Chromatin accessibility at the *Esr2*, *Ar*, and *Pgr* loci was low and selective across all clusters. Of these, for m33 we anticipated the greatest accessibility would be for *Esr2* as this has been reported to be expressed in GnRH neurons (102-104), although a recent report (105) suggests this transcript is not detectible in murine cells. Interestingly, accessibility was similar at *Pgr* and *Esr2*, being of medium intensity and in about half of GnRH neurons; the snRNAseq data indicated similar expression levels for these 2 genes but more GnRH neurons expressing *Pgr*, which has been detected in a subset of these cells (106). In contrast to the selective expression of the other sex steroid receptors, *Esr1* showed broad accessibility across nearly all clusters, as did the glucocorticoid receptor *Nr3c1*. This is of interest given the consistent the lack of detection of ERα protein in GnRH neurons (103, 107, 108) and the failure to detect *Esr1* transcript in individually harvested murine GnRH neurons in some (109), but not other (105), studies. The broad chromatin accessibility across diverse cell types suggests *Nr3c1* and *Esr1* transcriptional output in cells of the POA may be regulated more at the level of transcription factor action rather than chromatin availability. It also raises the possibility that, even in GnRH neurons, *Esr1* and *Nr3c1* chromatin may be poised for activation under certain physiological circumstances.

The FDR-supported differential accessibility findings reveal a pattern of cell-type-specific chromatin remodeling in PNA animals that spans both neuronal and non-neuronal populations. Two loci involve GnRH neurons, demonstrating prenatal androgen exposure results in differential accessibility in GnRH neurons even in the absence of coordinated transcriptional changes. One of these loci (chr2:986621718662171-986629888662988/*Gm10801*) was recently characterized as an enhancer; disruption of this locus is sufficient to drive a PCOS-like phenotype in *Greywick* mice (110). This region was also more accessible in clusters 16 (Glut/CCK) and 31 (GABA/BNC2), both neuronal subtypes that are implicated in eating behaviors, also dysregulated in *Greywick* mice. Among non-neuronal clusters, reduced *Pgk1* promoter accessibility across multiple glial cell types in PNA animals suggests PNA may alter the epigenetic regulation of glial energy metabolism. The VEH-enriched *Mdh2* promoter peak in GABAergic/Bnc2 neurons provides independent chromatin-level support for the oxidative metabolic differences suggested by GSEA, while PNA-enriched accessibility within an intronic region of *Ptprt* in GABAergic/Penk neurons points to potential remodeling of synaptic regulatory elements in a neuron population.

We pointed out limitations throughout this work, but it is useful to summarize several here. First, one sample was lost and another partially lost, leading to lower power and perhaps contributing to relatively few differences between treatments. Second, our design intentionally examined prepubertal mice before major phenotypic differences between VEH and PNA mice are observed to test if differences in GEX or chromosome accessibility occur before exposure to altered adult hormone milieu. While this addresses a specific question, it precludes examining differences that emerge in adulthood. Third, as the data had good LISI scores and other characteristics of being well integrated, we did not algorithmically force integration. This choice was made to preserve rare populations such as GnRH neurons, but it could affect interpretation. Fourth, doublets are reported but not removed. This includes 10 of 12 “rescued” putative GnRH neurons based on high GnRH GEX and an expression profile of other genes that resembled algorithmically identified GnRH neurons. Given evidence of context-dependent unreliability in doublet classification in this and other clusters and the strong marker gene-based support for GnRH identity in the rescued nuclei, we retained these nuclei in our primary reported GnRH population (*n* = 41). Of note, the (FDR = 0.08) upregulation of *Edil3* described above was detectable only when these putative doublets were included in the differential expression analysis and must be interpreted with caution pending independent confirmation. In contrast, the chromatin accessibility finding at the Gm10801/Greywick locus (chr2:98,662,1718662171-98,662,9888662988), while also sensitive in magnitude to inclusion of these nuclei, was independently significant across multiple clusters (16, 31). Fifth, pseudobulk-based testing across 4 biological replicates per condition is likewise underpowered relative to bulk approaches, but the *P*-value distributions for the RNA and ATAC comparisons showed a conservative, right-skewed pattern consistent with this limitation, such that surviving FDR-significant results could be regarded as a conservative subset of true differences. Finally, this is a hypothesis-generating data set. Further work is required to investigate other ages and to follow up on potential differences in pathways identified in this work.

GnRH neurons are a technically challenging neuronal population to study at single-cell resolution because they are few in number and sparsely distributed (67, 68). This challenge is illustrated by HypoMap (57), an integrated reference atlas combining 18 independent single-cell/single-nucleus datasets and nearly 385 000 cells across the murine hypothalamus. It has just 61 *Gnrh1*-expressing neurons after combining all constituent datasets. The 31 (algorithmic) or 41 (with manually identified) GnRH nuclei captured here represent a substantial addition. Nonetheless, rarity limits statistical power for differential expression and accessibility analyses and complicates automated doublet detection. The limited power, particularly for m33, may contribute to modest treatment effects, but biological explanations for possible temporal decoupling of chromatin accessibility changes from changes in transcription should also be considered. snATACseq describes where chromatin is physically available for the transcriptional and regulatory proteins to engage at single-cell resolution with minimal material. Accessibility, however, is only one layer of epigenetic regulation (111), note, for example, the unexpectedly high accessibility for *Esr1* and *Pgr* in GnRH neurons. Accessible chromatin is not a guarantee of active transcription: histone modifications such as H3K27me-mediated Polycomb repression can silence transcription even at nucleosome depleted loci, while DNA methylation at specific CpG sites can also block transcription factor binding or recruit repressors without broadly altering accessibility (112). Beyond chromatin, posttranscriptional regulation by non-coding RNAs operates on mRNA in the cytoplasm and may substantially shape the protein output of a given cell (113). The relative paucity of transcriptional differences at PND21 may itself be informative. Consistent with previous findings that prenatal androgen exposure alters the developmental trajectory of GnRH neurons and the broader neuroendocrine axis, these data suggest some consequences of prenatal androgen exposure are established early, but their functional manifestation emerges as the animal matures toward the full adult PCOS phenotype.

## Data Availability

Original data generated and analyzed during this study are included in this published article or in the data repositories listed in references (54, 65).
